# Differential effects of sex on longitudinal patterns of cognitive decline in Parkinson’s disease

**DOI:** 10.1007/s00415-020-10367-8

**Published:** 2021-01-05

**Authors:** Megan C. Bakeberg, Anastazja M. Gorecki, Jade E. Kenna, Alexa Jefferson, Michelle Byrnes, Soumya Ghosh, Malcolm K. Horne, Sarah McGregor, Rick Stell, Sue Walters, Paola Chivers, Samantha J. Winter, Frank L. Mastaglia, Ryan S. Anderton

**Affiliations:** 1 Perron Institute for Neurological and Translational Science, Nedlands, WA Australia; 2 Centre for Neuromuscular and Neurological Disorders, University of Western Australia, Nedlands, WA Australia; 3 School of Biological Sciences, University of Western Australia, Crawley, WA Australia; 4 Florey Institute for Neuroscience and Mental Health, University of Melbourne, Parkville, VIC Australia; 5 Centre for Clinical Neurosciences and Neurological Research, St Vincent’s Hospital Melbourne, Fitzroy, VIC Australia; 6 Institute for Health Research and School of Health Sciences, University of Notre Dame Australia, Fremantle, WA Australia; 7 Exercise Medicine Research Institute and School of Medical and Health Sciences, Edith Cowan University, Joondalup, WA Australia

**Keywords:** Parkinson’s disease, Sex, Cognitive decline, Domain specific, Longitudinal

## Abstract

**Background:**

Cognitive impairment is an important and diverse symptom of Parkinson’s disease (PD). Sex is a purported risk variable for cognitive decline in PD, but has not been comprehensively investigated.

**Objectives:**

This cross-sectional and longitudinal study examined sex differences in global and domain-specific cognitive performance in a large PD cohort.

**Methods:**

Cognitive function was evaluated using the Addenbrooke’s Cognitive Examination in 392 people with PD (PwP) from the Australian Parkinson’s Disease Registry. The influence of sex on domain-specific cognitive performance was investigated using covariate-corrected generalised linear models. In a repeated measures longitudinal subset of 127 PwP, linear mixed models were used to assess the impact of sex on cognition over time, while accounting for covariates.

**Results:**

Cross-sectional-corrected modelling revealed that sex was significantly predictive of cognitive performance, with males performing worse than females on global cognition, and memory and fluency domains. Longitudinally, sex was significantly predictive of cognitive decline, with males exhibiting a greater reduction in global cognition and language, whereas females showed a greater decline in attention/orientation, memory and visuospatial domains, despite starting with higher baseline scores. At follow-up, a significantly higher proportion of males than females fulfilled criteria for mild cognitive impairment or PD dementia.

**Conclusions:**

Sex was revealed as a significant determinant of overall cognitive performance as well as specific cognitive domains, with a differential pattern of decline in male and female participants. Such sex-specific findings appear to explain some of the heterogeneity observed in PD, warranting further investigation of mechanisms underlying this sexual dimorphism.

**Supplementary Information:**

The online version contains supplementary material available at 10.1007/s00415-020-10367-8.

## Introduction

Cognitive impairment is an often devastating and debilitating non-motor symptom of Parkinson’s disease (PD) [1, 2]. Upwards of 40% of people with Parkinson’s disease (PwP) develop some form of cognitive impairment, with a subset progressing from mild cognitive impairment (MCI) to a more severe Parkinson’s disease dementia (PDD) [1]. People often display executive dysfunction and attentional deficits in the early stages of the PD [3, 4] and progressively experience deficits in memory and visuospatial processing in the latter stages of the disease [3, 5]. However, cognitive deficits do not present in all PwP and can become apparent at any stage of the disease. Furthermore, the heterogeneity in both the presentation and rate of cognitive decline presents a challenge in the management and treatment of PD.

Studies examining demographic and clinical factors have identified increasing age, lower education levels, increasing disease duration, and motor symptom severity, among others, as contributing to susceptibility of cognitive impairment in PD [5]. Of these factors, patient sex not only influences disease risk [6, 7], but the presentation of several non-motor symptoms [8–10]. Sex is known to be an important factor when considering cognition [11–13], with sexual dimorphism observed across all ages in healthy populations [14–16]. Although sex associations within PD are well reported, there is a lack of studies investigating sex-specific effects on individual cognitive domains, as well as longitudinal cognitive domain-specific decline in PD. Prior studies examining the effect of sex have been cross sectional in nature [5, 9, 10, 12, 17–19], or have not considered the effect in various cognitive domains [20].

This study utilised a cohort of Australian PwP to assess the influence of sex on global and domain-specific cognitive performance. Further, a longitudinal subset of PwP was followed up to investigate the influence of sex on cognitive decline. It was thought that global and domain-specific sex differences in the architecture of cognitive ability and decline would be seen.

## Methods

### Participants

Three hundred and ninety-two home-based PwP (64.5% males, 35.5% females) were recruited sequentially into the Australian Parkinson’s Disease Registry (APDR) as previously described [8, 21]. In brief, participants were enrolled from Movement Disorders Clinics at the Perron Institute for Neurological and Translational Science (Perth, Western Australia), St. Vincent’s Hospital (Melbourne, Victoria), and Royal North Shore Hospital (Sydney, New South Wales), between 2012 and 2019. Individuals with a prior diagnosis of dementia, diffuse Lewy body disease, or other neurological disorders or disabling medical conditions were excluded. All PwP were examined by movement disorder neurologists prior to inclusion in the study for verification of the diagnosis in accordance with the UK Brain Bank criteria for idiopathic PD [22]. For longitudinal studies, a subset of participants (*n* = 127, 62% males, 37% females) were followed up for between 1 and 7 years following initial assessment. This subsection of participants did not include those enrolled through the Royal North Shore Hospital, though did include those participants who were still able to attend Movement Disorder Clinics in Perth and Melbourne. This study was approved by the Human Research and Ethics Committees (Approval number 2006/073 and Approval number RA/4/20/4470). Written informed consent was obtained from all participants, in accordance with the Australian National Health and Medical Research Council guidelines.

### Clinical assessments of PwP

Clinical evaluations included collection of patient demographic variables and medication dosage, assessments of motor and cognitive function, and other disease-related features (Table 1). Parkinsonian medications were converted to a total levodopa equivalent daily dose (LEDD), based on a previously reported conversion equation [23], 24]. Motor symptoms were evaluated in the ‘ON’ state using the Movement Disorder Society-Unified Parkinson's Disease Rating Scale (MDS-UPDRS) Part III, and Hoehn and Yahr (H&Y) Scale [25].

**Table 1:** Clinical characteristics of the cross-sectional PD cohort (*n* = 392)

Clinical characteristics	Mean (SD) or *n* (%)
Age at assessment (years)	64.8 (9.2)
Age at onset (years)	56.9 (10.2)
Disease duration (years)	7.9 (5.7)
Sex	
Male	253 (64.5%)
Female	139 (35.5%)
Medication naïve	
Yes	32 (8.2%)
No	360 (91.8%)
LEDD (mg/day)	889.5 (579.6)
DBS	
Yes	40 (10.2%)
No	352 (89.8%)
H&Y	1.8 (0.9)

*SD* standard deviation, *%* percentage, *LEDD* levodopa equivalent daily dose, *DBS* deep brain stimulation, *MDS-UPDRS III* Movement Disorder Society-Unified Parkinson’s Disease Rating Scale III, *H&Y* Hoehn & Yahr

### Cognitive testing

Each participant was evaluated by a trained cognition researcher and completed a panel of standardised neuropsychological assessments, as previously described [8, 21]. In addition, cognitive function was assessed in this study using the revised ‘Addenbrooke’s Cognitive Examination’ (ACE-R). The ACE-R is a brief, 20-min screening battery, which provides an evaluation of global cognitive function (total possible score of 100), as well as domain-specific assessment of attention, orientation, memory, verbal fluency, language, visuospatial and perceptual abilities [26, 27]. In similar cohorts to the current cohort, pre-determined cut-off ACE-R scores of ≤ 88 and ≤ 82 have been validated as markers of MCI [28] and PDD [26], respectively. In the current cohort, as previously determined in academic literature, these scores were used to indicate probable cases of MCI and PDD, respectively. Though these were not taken as diagnostic of MCI nor PDD.

### Statistical methods

Data were analysed using IBM SPSS (version 26, IBM Corporation). Variables were described using mean and standard deviation (in brackets, SD), or frequency and percent (in brackets, %), as appropriate. Continuous variable distributions were assessed using the Shapiro–Wilk test of normality. Sex differences for clinical characteristics were assessed using independent *t* tests (or non-parametric Mann–Whitney *U* test) or Chi square. A significant nominal *p* value of < 0.05 was employed for all statistical tests.

For cross-sectional analysis, naïve generalised linear models (GLM) were used as univariate models to assess whether patient clinical characteristics were associated with total and sub-scale cognitive scores, and were, therefore, covariates of cognition. Variables included age at time of first assessment, age at disease onset, disease duration, LEDD and deep brain stimulation (DBS) history. Thus, variables identified as being statistically significant in univariate models were considered covariates and were included in the multivariable-corrected GLMs. Such corrected models assessed the impact of sex on cognitive performance while accounting for covariates.

For longitudinal studies, trends of mean clinical assessments and patient clinical characteristics were assessed over time using generalised linear mixed models (GLMMs). Naïve GLMMs were used as univariate models to assess whether the patient clinical characteristics were significantly associated with cognitive total and sub-scale scores over-time, and were, therefore, covariates of cognitive decline. Variables assessed included years between assessments, age at assessment, age at disease onset, disease duration, LEDD and DBS history. Finally, variables identified as being statistically significant in univariate models were considered covariates and were included in the multivariable-corrected GLMMs. Such corrected models were constructed to study the effect of sex on cognition over time, whilst controlling for covariates previously identified as being risk factors for the progression of cognitive impairment in PD.

Akaike information criterion (AIC) was used to compare model fit, where a lower value indicated better model fit. Residual plots were examined for all models and no violations were noted. To evaluate the association between sex and progression to probable MCI or PDD, two methods were used. First, taking the “onset” of MCI or PDD as the endpoint, survival curves for males and females were estimated by the Kaplan–Meier method. To compare the survival curves, the log rank test was applied, placing weight on longer survival periods [29, 30]. Second, grouped participants with a follow-up period of more than 3 years (*n* = 70) were analysed using Chi square and binary logistic regression for naïve and corrected models, respectively (covariates included in corrected models identified in Supplementary Tables 1, 3).

## Results

### Demographics and clinical details of cross-sectional cohort

Clinical and demographic information for the PD cohort at baseline is presented in Table 1. The cohort was predominated by males (64.5%), with participants having a mean age of 64.8 (9.2) years and a mean disease duration of 7.9 (5.7) years. In this instance, 32 individuals were naïve of traditional parkinsonian medication, while in those on treatment, the average LEDD was 889.5 (579.6) mg/day. Furthermore, 10.2% of participants had undergone DBS, and participants had an average H&Y score of 1.8 (0.9). Overall, the cohort exhibited an average total ACE-R score of 88.89 (10.83) (Table 2). Statistically significant sex differences were not observable between age at assessment, nor age of onset, nor disease duration. However, females were found to have significantly lower LEDD doses (784.49 mg/day) than males (949.52 mg/day; *p* = 0.008), and a higher proportion of males had undergone DBS (12.6%) than females (5.8%; *p* = 0.017). Therefore, these variables were included in further statistical analyses. The relationship between cognitive scores and clinical characteristics is shown in Supplementary Table 1, which shows that total ACE-R scores, as well as all subdomain scores, were significantly associated with age at assessment and disease duration.

**Table 2:** Significance of sex differences in naïve and corrected linear regression models for ACE-R total and sub-domain scores

ACE-R variable	Total mean (*n* = 392)^a^	Male mean (*n* = 253)^a^	Female mean (*n* = 139)^a^	Naïve (*p* value)^b^	Covariate corrected (*p* value)
Total ACE-R	88.89 (10.83)	88.24 (10.53)	90.07 (11.29)	0.098	**0.044**^**c**^
Attention and orientation	17.37 (1.59)	17.28 (1.61)	17.53 (1.54)	0.143	0.289^d^
Memory	22.26 (3.77)	21.93 (3.96)	22.85 (3.33)	**0.017**	**0.030**^**e**^
Fluency	9.98 (3.33)	9.60 (3.55)	10.66 (2.77)	**0.002**	**0.006**^**f**^
Language	24.82 (1.94)	24.65 (2.11)	25.12 (1.56)	**0.018**	0.067^g^
Visuospatial-perceptual	14.66 (2.49)	14.55 (2.72)	14.85 (1.98)	0.253	0.685^h^

^a^Data are presented as mean raw scores (SD)

^b^*p* value taken from GLM without correction for covariates

^c^Corrected for age at assessment, age at onset, disease duration and LEDD, identified in Supplementary Table 1

^d^Corrected for age at assessment, disease duration, LEDD and DBS status, identified in Supplementary Table 1

^e^Corrected for age at assessment, age at onset, disease duration and DBS status, identified in Supplementary Table 1

^f^Corrected for age at assessment, disease duration and DBS status, identified in Supplementary Table 1

^g^Corrected for age at assessment, age at onset, disease duration and LEDD, identified in Supplementary Table 1

^h^Corrected for age at assessment, disease duration and DBS status, identified in Supplementary Table 1

ACE-R, Addenbrooke’s Cognitive Examination—Revised; SD, standard deviation

### Discriminatory effect of sex on cross-sectional cognitive scores

Naïve GLMs revealed that sex was significantly associated with ACE-R subdomain scores, namely memory (*p* = 0.017), fluency (*p* = 0.002) and language (*p* = 0.018) (Table 2). Univariate analysis (naïve GLMs) was completed to determine demographic and clinical variables associated with ACE-R total and sub-domain scores (Supplementary Table 1). Significantly associated variables were treated as covariates and incorporated into corrected multivariable models investigating the role of sex in predicting cross-sectional cognitive scores. Multivariable GLMs indicated that sex was significantly predictive of global cognitive performance, as measured by total ACE-R (*p* = 0.044), as well as subdomain scores for memory (*p* = 0.030) and fluency (*p* = 0.006), when controlling for covariates (Table 2). AIC for each model can be found in Supplementary Table 2, demonstrating corrected models as being a better fit than naïve models. Males exhibited a mean total ACE-R score of 1.83 points lower than females, 0.92 points lower in the memory subdomain than their female counterparts, and 1.06 points lower in the fluency subdomain (Table 2).

### Longitudinal cohort information and clinical data

Clinical and demographic information for the longitudinal sub-cohort at baseline and follow-up is presented in Table 3. This sub-cohort remained predominated by males (63.0%), with participants having an average age of 63.1 (9.5) years and a disease duration of 6.7 (5.1) years at baseline. Mean time between assessments was 3.0 (1.9) years, which was consistent with mean age and disease duration for follow-up assessments (Table 4). At the time of initial assessment 11 individuals were drug-naive, with five (5) of those individuals having begun anti-parkinsonian medications by the time of the follow-up assessment. In those who were on medication, the average LEDD was 829.3 (565.3) mg/day at initial assessment and 886.6 (629.7) mg/day at follow-up. Ten (10) participants were having DBS at baseline, and a further six (6) underwent the procedure during the follow-up period. Overall, the cohort exhibited an average total ACE-R score of 90.1 (8.1) at baseline which was reduced by 2.4 points over the assessment period. Mean ACE-R subdomain scores at both assessment points can be seen in Table 4.

**Table 3:** Baseline and follow-up clinical characteristics of the longitudinal PD sub-cohort (n = 127)

Clinical characteristics	Mean (SD) or *n* (%)	
	Baseline	Follow-up
Follow-up interval (years)	–	3.0 (1.9)
Age at assessment (years)	63.1 (9.5)	66.1 (9.2)
Age at onset (years)	56.5 (10.6)	56.5 (10.6)
Disease duration (years)	6.7 (5.1)	9.6 (5.7)
Sex		
Male	80 (63.0%)	80 (63.0%)
Female	47 (37.0%)	47 (37.0%)
Medication naïve		
Yes	11 (8.7%)	6 (4.7%)
No	116 (91.3%)	121 (95.3%)
LEDD (mg/day)	829.3 (565.3)	886.6 (629.7)
DBS		
Yes	10 (7.9%)	16 (12.6%)
No	117 (92.1%)	111 (87.4%)
H&Y	1.6 (0.9)	2.3 (0.9)
MDS-UPDRS III	17.8 (13.8)	26.3 (23.3)
ACE-R score		
Total	90.1 (8.1)	87.7 (11.3)
Attention and orientation	17.4 (1.4)	17.2 (1.6)
Memory	22.1 (3.7)	21.1 (4.5)
Fluency	10.3 (3.1)	10.9 (2.9)
Language	25.2 (1.3)	24.1 (2.8)
Visuospatial-perceptual	14.9 (1.9)	14.4 (2.4)

*SD* standard deviation, *%* percentage, *LEDD* levodopa equivalent daily dose, *DBS* deep brain stimulation, *MDS-UPDRS III* Movement Disorder Society-Unified Parkinson’s Disease Rating Scale III, *H&Y* Hoehn & Yahr, *ACE-R* Addenbrooke’s Cognitive Examination—Revised

**Table 4:** Significance of sex differences in naïve and corrected generalised linear mixed models for total ACE-R total and sub-domain scores over time

ACE-R	Males at baseline (*n* = 80)^a^	Females at baseline (*n* = 47)^a^	Males at follow-up (*n* = 80)^a^	Females at follow-up (*n* = 47)^a^	Naïve (*p* value)^b^	Corrected (*p* value)
Total ACE-R	89.00 (8.56)	91.85 (6.99)	85.98 (12.53)	90.48 (6.99)	**0.005**	**0.001**^**c**^
Attention and orientation	17.26 (1.28)	17.64 (1.44)	17.04 (1.89)	17.34 (1.03)	0.069	**0.049**^**d**^
Memory	21.49 (3.77)	23.00 (3.51)	20.59 (4.85)	21.91 (3.73)	**0.006**	**0.002**^**e**^
Fluency	10.06 (3.44)	10.77 (2.53)	10.49 (2.99)	11.70 (2.44)	**0.011**	**0.004**^**f**^
Language	25.05 (1.31)	25.32 (1.14)	23.72 (3.23)	24.49 (1.71)	0.064	**0.040**^**g**^
Visuospatial-perceptual	14.71 (2.31)	15.40 (1.12)	14.21 (2.75)	14.72 (1.79)	**0.028**	**0.011**^**h**^

^a^Data are presented as mean raw scores (SD)

^b^*p* value taken from GLMM without correction for covariates

^c^Corrected for age at assessment and age at onset, as identified in Supplementary Table 3

^d^Corrected for age at assessment and age at onset, as identified in Supplementary Table 3

^e^Corrected for age at assessment and age at onset, as identified in Supplementary Table 3

^f^Corrected for age at assessment and age at onset, as identified in Supplementary Table 3

^g^Corrected for years between assessments, age at assessment and age at onset, as identified in Supplementary Table 3

^h^Corrected for years between assessments, age at assessment and age at onset, as identified in Supplementary Table 3

*ACE-R* Addenbrooke’s cognitive examination—revised, *SD* standard deviation

### Sex is predictive of performance in all cognitive domains over time

Naïve GLMMs revealed that sex was significantly associated with ACE-R total score (*p* = 0.005) as well as selective subdomain ACE-R assessment scores, namely memory (*p* = 0.006), fluency (*p* = 0.011) and visuospatial-perceptual (*p* = 0.028) (Table 4). Univariate analysis (naïve GLMs) was carried out to determine which demographic and clinical characteristics were associated with ACE-R total and subdomain scores (Supplementary Table 3). Significantly associated variables were treated as covariates and incorporated into corrected multivariable models to investigate the predictive role of sex in longitudinal cognitive decline. Multivariable GLMMs indicated that sex was significantly predictive of global cognition, as measured by total ACE-R (*p* = 0.001), as well as all subdomain scores assessed (Table 4). AIC for each model can be found in Supplementary Table 4, exhibiting corrected models as being a better fit than naïve models in each instance.

Females exhibited higher mean baseline and follow-up scores across all facets of cognition examined, though to a varying degree dependent on which subdomain of cognition is considered (Table 4). However, the degree of change from baseline to follow-up was varied amongst cognitive domains and based on sex. Males experienced a reduction in mean global cognition of 3.02 points, compared to 1.37 points in females. Similarly, in the language domain, males experienced a mean reduction of 1.3 points, 0.5 points more than that experienced by females. On the other hand, as shown in Table 4, the decline in attention and orientation, memory, and visuospatial-perceptual domains was greater in females than in males (Table 4).

### Higher proportion of males progress to potential MCI or PDD over time

**Fig. 1:**
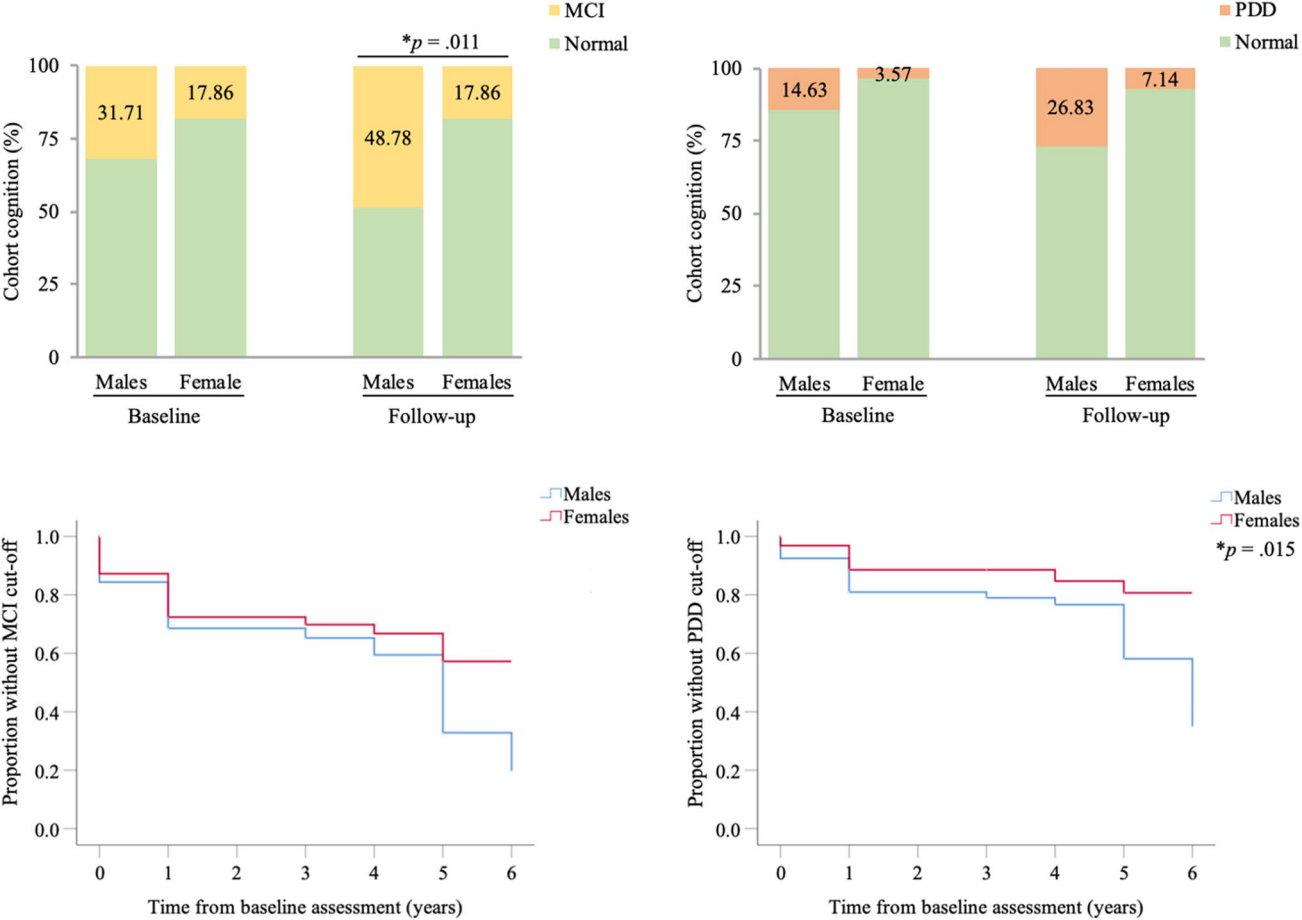
Participants classified as MCI (**a**) and PDD (**b**) over time, corrected for age at assessment and age at onset. Kaplan–Meier curves depicting the effect of sex on the proportion of participants classified as MCI (**c**) and PDD (**d**). *%* percentage, *MCI* mild cognitive impairment, *PDD* Parkinson’s disease dementia

Following this, participants were grouped based on the pre-determined ACE-R cut-off scores, which were taken as indicative of a probable case of MCI or PDD, though not a definitive diagnostic marker. In exploring grouped comparisons, it was seen that the percentage of males and females classified as having scores reflective of potential MCI or PDD did not differ significantly between the sexes at baseline. However, the percentage of males potentially classifiable as having MCI at follow-up was significantly higher than females (Fig. 1a, Naïve *p* = 0.009, Corrected *p* = 0.011). At baseline, 31.71% of males were considered to have a probable case of MCI, whereas at follow-up nearly 50% of males exhibited cognitive scores to warrant a probable MCI diagnosis (Fig. 1a). In addition, the percentage of males classified as having scores likely indicative of PDD at follow-up was also significantly higher than females following naïve assessment (Fig. 1b, *p* = 0.040), but not following covariate correction (Fig. 1b, *p* = 0.078). Survival analysis, taking “MCI” or “PDD” onset as the endpoints, exhibited differences between males and females (Fig. 1c and d). There was a higher proportion of males progressing to what may be classifiable as MCI, though this was not statistically significant (Fig. 1c, Log rank *p* = 0.057). There was, however, a significant proportion of males progressing to what may be classifiable as PDD when compared to females (Fig. 1d, log rank *p* = 0.015).

## Discussion

The occurrence of cognitive impairment is known to be an important determinant of morbidity and impaired quality of life in PwP, as well as having a major impact on caregiver burden. Furthermore, the heterogeneity of cognitive impairment risk and varied trajectory of cognitive decline with disease progression represents a difficult barrier in understanding and managing this facet of PD. Importantly, studies have noted sex as a risk variable in the onset of cognitive impairment, both in PD and in other populations. However, there have been few previous comprehensive investigations of sex-based differences in individual cognitive domains over time [31], as in the present study. Some prior studies have been purely cross sectional in nature [5, 9, 10, 12, 17–19], while longitudinal studies have not examined the sex-specific differences in various cognitive domains [20], or have not focussed primarily on elucidating the impact of sex on cognition [32]. Here, our findings suggest that sex is a significant determinant of domain-specific cognitive performance, in agreement with prior literature. Furthermore, we show that sex is a significant predictor of cognitive decline over time, and of the likelihood of progressing to what may be considered a likely case of MCI. Interestingly, the proportion of females with cognitive scores that may potentially warrant a clinical diagnosis of MCI did not change longitudinally, highlighting the role of investigating cognition at the domain-specific level. Such sex-specific findings appear to explain some of the heterogeneity observed in the cognitive symptomatology of the disease, warranting further investigation into the underlying mechanisms of this sexual dimorphism.

Cross-sectional analysis in the present cohort revealed an association between sex and cognition, with males performing significantly worse than females in global cognition and in memory and fluency subdomains. Literature has reported sex differences in patterns of cognition in healthy young adults (ages 18–31) [15], as well as in adulthood and the ageing population [16]. In recent studies, young females were found to exhibit superior nonverbal reasoning skills and faster language decision-making than their male counterparts [14, 15], a finding that has also been reflected in older cohorts [16]. To explain these differences, neuroimaging studies have reported divergent patterns of brain connectivity, with males exhibiting a greater connectivity between the left superior parietal lobule (SPL) and posterior superior temporal gyrus (STG), whereas females had a greater connectivity between the left inferior frontal gyrus (IFG) and posterior STG [15, 33]. Furthermore, it was apparent that sex differences existed in inhibitory connections in these same regions. Apart from the SPL, such regions are typically associated with semantic fluency and language ability. The left posterior STG specifically contributes to the ability to produce sounds that form words (lexical phonology), as well as language comprehension and working memory [34, 35], and the functional connection between the IFG and posterior STG is involved in speech and language processing [36]. Whereas, the SPL is thought to be involved in spatial and visual perception [34]. These sex differences in structural and functional connectedness and networks may provide an explanation for the current findings of sex-specific changes in cognitive performance [13], whereby females had superior performance in fluency and memory. For instance, in the context of Parkinson’s disease, a number of the aforementioned structures have been implicated in patients with MCI and PDD [37, 38]. Notably, it has been reported that PwP who have MCI are more likely to have cortical thinning, particularly in the STG [39, 40]. Moreover, findings from the CamPaIGN study indicate that fluency tasks, which recruit structures of the temporal lobe, are associated with clinical presentation of cognitive impairment over time [5], results that are also reflected in the current cohort.

When examining cognition changes longitudinally in PwP, sex appeared to be a significant predictor of cognitive ability over time regardless of which domain was assessed. Overall, in global cognitive measures, males were noted to decline significantly more than females. Furthermore, it was observable that a higher proportion of males progressed to what could be considered as MCI, a clinically recognisable form of cognitive impairment. Such findings are reflective of current literature [9, 10, 17, 20]. Researchers have cited the neuroprotective effects of oestrogen as potentially playing a role in this effect [11, 13]. Not only have oestrogens been found to play a role in dopaminergic neurodegeneration in PD, but the hormone has been found to have favourable effects on neuroinflammation, oxidative stress and iron metabolism within the context of PD [41]. Furthermore, sex differences in microglial and astrocytic cells, such as their heightened sensitivity to inflammatory stimuli [42–44] and their anatomical distribution [45, 46], have been postulated to mediate sex differences in cognition and memory [47, 48]. It is worthwhile considering that such results may be reflective of the superior baseline performance of females in cognitive measures than males or may be exacerbated by disease process, though prior research has found that sex-specific progression to cognitive impairment cannot be fully explained by this baseline performance, nor disease duration [31].

While previous studies have focussed on progression to a clinically diagnosable form of cognitive impairment, namely MCI or PDD, our findings point to noteworthy domain-specific effects of sex over time. Though males appeared to perform worse overall, what did vary between facets of cognition was which sex was protected, the directionality of cognitive ability, and the rate of decline. For instance, despite females exhibiting consistently superior overall cognitive performance at baseline, females had a greater decline in attention and orientation, memory, and visuospatial-perceptual ability over time, compared to males. Whereas, males had a greater decline in the language domain than females. These findings suggest that there is a differential sex-effect on domain-specific cognitive decline, but it remains to be determined what the implications of these differential patterns of decline are for progression to dementia. Though studying clinically distinguishable MCI and PDD is of importance, we see here that females do not progress to a state that may be indicative of MCI or PDD to the same extent as males; however, they do experience a greater decline in certain domains of their cognition than males. Such sex-specific effects on the architecture of cognitive decline may be of importance, and have not always been considered in the past. It is of particular interest because the decline seen in females here is in spite of their higher baseline scores and the putative neuroprotective effect of oestrogen. The role that this hormone plays in the sexual dimorphism reported in this study, predominantly in regard to the domain-specific rate and directionality of cognitive ability over time, remains elusive and represents an area which requires further investigation.

## Limitations

A number of limitations of the current study must be noted. First, at baseline, home-based PwP were recruited sequentially from three different movement disorder centres across Australia, which could have had the potential of introducing scoring variability. However, as the clinical assessments were performed by trained clinician-researchers this is unlikely to have had a significant impact on the data. Second, initial recruitment excluded individuals with more advanced PD and dementia, which is likely to have contributed to a lower mean age of symptom onset and higher cognitive scores at baseline, and the cohort is, therefore, not truly representative of a community-based sample. Further to this, follow-up recruitment were not able to include all baseline participants, in part this was due to the advanced nature of disease severity, which may introduce a degree of bias. In addition, the cohort was not recruited based upon sex, which has resulted in an uneven balance of male and female participants and may have altered findings. However, the proportion of males to females in the study is reflective of PD incidence in prior literature. In turn, despite being a contributor to cognitive functioning, education levels were not able to be obtained. Thus, were not included in statistical analyses. Finally, as the ACE-R includes a paucity of tests of executive function [27]. Therefore, the present findings should be confirmed using other more comprehensive cognitive testing protocols in larger longitudinal studies, while taking into consideration confounding variables that were not able to be included in this manuscript.

## Conclusion

Here we report that sex is a significant determinant of cognitive decline in PD, both cross sectionally and in a repeated measures longitudinal study. Males were seen to perform significantly worse when considering disease course over time, and showed a greater likelihood than females of progressing to a probable case of MCI and PDD. However, it was apparent that in spite of maintaining a higher overall level of cognitive performance, females did experience a selective decline in certain cognitive domains with disease progression, highlighting the importance of examining for subclinical cognitive impairment. While the extent to which the differential effects of disease progression are responsible for the observed sex differences in cognition remains unclear, it is likely that PD-related cortical and subcortical pathology, coupled with age-related changes, may exacerbate sex differences in the severity and trajectory of cognitive decline. Overall, the results from this study support the consideration of sex in explaining some of the clinical heterogeneity observed in PD-related cognitive decline. Better understanding of the role of sex in the landscape of cognitive decline may help in stratifying different patterns of cognitive impairment and aid in the development of individualised treatment strategies for PwP.

## Supplementary Information

Below is the link to the electronic supplementary material.

Supplementary file1 (DOCX 28 KB)

## Data Availability

Anonymised data are available upon reasonable request from qualified investigators.

## References

[1] Litvan I, Goldman JG, Troster AI, Schmand BA, Weintraub D, Petersen RC (2012). Diagnostic criteria for mild cognitive impairment in Parkinson’s disease: movement Disorder Society Task Force guidelines. Mov Disord.

[2] Santangelo G, Vitale C, Picillo M, Moccia M, Cuoco S, Longo K (2015). Mild cognitive impairment in newly diagnosed Parkinson’s disease: a longitudinal prospective study. Parkinsonism Relat Disord.

[3] Aarsland D, Bronnick K, Williams-Gray C, Weintraub D, Marder K, Kulisevsky J (2010). Mild cognitive impairment in Parkinson disease: a multicenter pooled analysis. Neurology.

[4] Muslimovic D, Post B, Speelman JD, Schmand B (2005). Cognitive profile of patients with newly diagnosed Parkinson disease. Neurology.

[5] Williams-Gray CH, Evans JR, Goris A, Foltynie T, Ban M, Robbins TW (2009). The distinct cognitive syndromes of Parkinson’s disease: 5 year follow-up of the CamPaIGN cohort. Brain.

[6] Baldereschi M, Di Carlo A, Rocca WA, Vanni P, Maggi S, Perissinotto E (2000). Parkinson’s disease and parkinsonism in a longitudinal study: two-fold higher incidence in men. ILSA Working Group. Italian Longitudinal Study on Aging. Neurology.

[7] Van Den Eeden SK, Tanner CM, Bernstein AL, Fross RD, Leimpeter A, Bloch DA (2003). Incidence of Parkinson’s disease: variation by age, gender, and race/ethnicity. Am J Epidemiol.

[8] Riley M, Bakeberg M, Byrnes M, Jefferson A, Ghosh S, Stell R (2018). Demographic and clinical predictors of trait impulsivity in Parkinson’s disease patients. Parkinsons Dis.

[9] Nicoletti A, Vasta R, Mostile G, Nicoletti G, Arabia G, Iliceto G (2017). Gender effect on non-motor symptoms in Parkinson’s disease: are men more at risk?. Parkinsonism Relat Disord.

[10] Szewczyk-Krolikowski K, Tomlinson P, Nithi K, Wade-Martins R, Talbot K, Ben-Shlomo Y, et al. (2014) The influence of age and gender on motor and non-motor features of early Parkinson’s disease: Initial findings from the Oxford Parkinson Disease Center (OPDC) discovery cohort 10.1016/j.parkreldis.2013.09.025 24183678

[11] Miller IN, Cronin-Golomb A (2010). Gender differences in Parkinson’s disease: clinical characteristics and cognition. Mov Disord.

[12] Augustine EF, Pérez A, Dhall R, Umeh CC, Videnovic A, Cambi F (2015). Sex differences in clinical features of early, treated Parkinson’s Disease Nazir A, editor. PLoS ONE.

[13] Lin S-J, Baumeister TR, Garg S, McKeown MJ (2018) Cognitive profiles and hub vulnerability in Parkinson’s Disease. Front Neurol 9 10.3389/fneur.2018.00482 PMC6019441 29973913

[14] Satterthwaite TD, Wolf DH, Roalf DR, Ruparel K, Erus G, Vandekar S (2015). Linked sex differences in cognition and functional connectivity in Youth. Cereb Cortex.

[15] Xu M, Liang X, Ou J, Li H, Luo Y, Tan LH (2019) Sex differences in functional brain networks for language. Cereb Cortex 10.1093/cercor/bhz184 31512720

[16] Li R, Singh M (2014). Sex differences in cognitive impairment and Alzheimer’s disease. Front Neuroendocrinol.

[17] Liu R, Umbach DM, Peddada SD, Xu Z, Tröster AI, Huang X, et al. (2015) Potential sex differences in nonmotor symptoms in early drug-naive Parkinson disease 10.1212/WNL.0000000000001609 PMC4451049 25925983

[18] Song Y, Gu Z, An J, Chan P (2014). Gender differences on motor and non-motor symptoms of de novo patients with early Parkinson’s disease. Neurol Sci.

[19] Reekes TH, Higginson CI, Ledbetter CR, Sathivadivel N, Zweig RM, Disbrow EA (2020). Sex specific cognitive differences in Parkinson disease. npj Park Dis.

[20] Cholerton B, Johnson CO, Fish B, Quinn JF, Chung KA, Peterson-Hiller AL (2018). Sex differences in progression to mild cognitive impairment and dementia in Parkinson’s disease. Parkinsonism Relat Disord.

[21] Bakeberg MC, Jefferson A, Riley M, Byrnes M, Ghosh S, Mastaglia FL (2019). Elevated serum homocysteine levels have differential gender-specific associations with motor and cognitive states in Parkinson’s Disease. Parkinsons Dis.

[22] Hughes AJ, Daniel SE, Kilford L, Lees AJ (1992). Accuracy of clinical diagnosis of idiopathic Parkinson’s disease: a clinico-pathological study of 100 cases. J Neurol Neurosurg Psychiatry.

[23] Parkin SG, Gregory RP, Scott R, Bain P, Silburn P, Hall B (2002). Unilateral and bilateral pallidotomy for idiopathic Parkinson’s disease: a case series of 115 patients. Mov Disord.

[24] Tomlinson CL, Stowe R, Patel S, Rick C, Gray R, Clarke CE (2010). Systematic review of levodopa dose equivalency reporting in Parkinson’s disease. Mov Disord.

[25] Goetz CG, Fahn S, Martinez-Martin P, Poewe W, Sampaio C, Stebbins GT (2007). Movement disorder society-sponsored revision of the Unified Parkinson’s Disease Rating Scale (MDS-UPDRS): process, format, and clinimetric testing plan. Mov Disord.

[26] Mioshi E, Dawson K, Mitchell J, Arnold R, Hodges JR (2006). The Addenbrooke’s cognitive examination revised (ACE-R): a brief cognitive test battery for dementia screening. Int J Geriatr Psychiatry.

[27] Bakeberg MC, Riley M, Byrnes M, Mastaglia FL, Anderton RS (2020). Clinically assessing cognitive function in Parkinson’s disease. Diagnosis and Management of Parkinson’s disease. Diagnosis Manag. Park. Dis..

[28] Marras C, Armstrong MJ, Meaney CA, Fox S, Rothberg B, Reginold W (2013). Measuring mild cognitive impairment in patients with Parkinson’s disease. Mov Disord.

[29] Inzelberg R, Bonuccelli U, Schechtman E, Miniowich A, Strugatsky R, Ceravolo R (2006). Association between amantadine and the onset of dementia in Parkinson’s disease. Mov Disord.

[30] Papadimitriou D, Antonelou R, Miligkos M, Maniati M, Papagiannakis N, Bostantjopoulou S (2016). Motor and nonmotor features of carriers of the p.A53T Alpha-Synuclein Mutation: a longitudinal study. Mov Disord.

[31] Iwaki H, Blauwendraat C, Leonard HL, Makarious MB, Kim JJ, Liu G, et al. (2020) Differences in the presentation and progression of Parkinson’s Disease by Sex. Mov Disord 10.1002/mds.28312 PMC7883324 33002231

[32] Locascio JJ, Corkin S, Growdon JH (2003). Relation between clinical characteristics of Parkinson’s disease and cognitive decline. J Clin Exp Neuropsychol.

[33] Gong G, Rosa-Neto P, Carbonell F, Chen ZJ, He Y, Evans AC (2009). Age- and gender-related differences in the cortical anatomical network. J Neurosci.

[34] Graves WW, Grabowski TJ, Mehta S, Gupta P (2008). The left posterior superior temporal gyrus participates specifically in accessing lexical phonology. J Cogn Neurosci.

[35] Friederici AD (2011). The brain basis of language processing: from structure to function. Physiol Rev.

[36] Garell PC, Bakken H, Greenlee JDW, Volkov I, Reale RA, Oya H (2013). Functional connection between posterior superior temporal gyrus and ventrolateral prefrontal cortex in human. Cereb Cortex.

[37] Guimarães RP, Santos MCA, Dagher A, Campos LS, Azevedo P, Piovesana LG, et al. (2016) Pattern of reduced functional connectivity and structural abnormalities in Parkinson’s Disease: an exploratory study. Front Neurol 7 10.3389/fneur.2016.00243 PMC5233672 28133455

[38] Hanganu A, Monchi O (2016) Structural neuroimaging markers of cognitive decline in Parkinson’s Disease. Parkinsons Dis 10.1155/2016/3217960 PMC4848447 27190672

[39] Lewis MM, Du G, Lee E-Y, Nasralah Z, Sterling NW, Zhang L (2016). The pattern of gray matter atrophy in Parkinson’s disease differs in cortical and subcortical regions. J Neurol.

[40] Hanganu A, Bedetti C, Degroot C, Mejia-Constain B, Lafontaine A-L, Soland V (2014). Mild cognitive impairment is linked with faster rate of cortical thinning in patients with Parkinson’s disease longitudinally. Brain.

[41] Cerri S, Mus L, Blandini F (2019). Parkinson’s disease in women and men: what’s the difference?. J Parkinsons Dis.

[42] Villa A, Gelosa P, Castiglioni L, Cimino M, Rizzi N, Pepe G (2018). Sex-specific features of microglia from adult mice. Cell Rep.

[43] Siani F, Greco R, Levandis G, Ghezzi C, Daviddi F, Demartini C (2017). Influence of estrogen modulation on glia activation in a murine model of Parkinson’s Disease. Front Neurosci.

[44] Martin-Jiménez C, Gaitán-Vaca DM, Areiza N, Echeverria V, Ashraf GM, González J (2019). Astrocytes mediate protective actions of estrogenic compounds after traumatic brain injury. Neuroendocrinology.

[45] Schwarz JM, Sholar PW, Bilbo SD (2012). Sex differences in microglial colonization of the developing rat brain. J Neurochem.

[46] Lenz KM, Nugent BM, Haliyur R, McCarthy MM (2013). Microglia are essential to masculinization of brain and behavior. J Neurosci.

[47] Yun J, Yeo IJ, Hwang CJ, Choi D-Y, Im H-S, Kim JY (2018). Estrogen deficiency exacerbates Aβ-induced memory impairment through enhancement of neuroinflammation, amyloidogenesis and NF-ĸB activation in ovariectomized mice. Brain Behav Immun.

[48] Chamniansawat S, Sawatdiyaphanon C (2018). Age-related memory impairment associated with decreased endogenous estradiol in the hippocampus of female rats. Int J Toxicol.

