# Supplementary material for: Differential effects of sex on longitudinal patterns of cognitive decline in Parkinson’s disease
Source: J Neurol. 2021 Jan 5;268(5):1903–12. doi: 10.1007/s00415-020-10367-8 (PMC8068663; doi:10.1007/s00415-020-10367-8)
Supplement: Supplementary file 1 — Supplementary file1 (DOCX 28 KB) [file 415_2020_10367_MOESM1_ESM.docx]

**Differential Effects of Sex on Longitudinal Patterns of Cognitive Decline in Parkinson’s disease**

Megan C. Bakeberg, BBMSc(Hons) ^1,2^, Anastazja M. Gorecki, BSc(Hons)^1,3^, Jade E. Kenna BSc(Hons)^1,2^, Alexa Jefferson, BN^1^, Michelle Byrnes, MPsych, PhD^1,2^, Soumya Ghosh, MBBS, PhD, FRACP^1,2^, Malcolm K. Horne, MBBS, PhD, FRACP^4,5^, Sarah McGregor BSc(Hons)^5^, Rick Stell, MBBS, RACP^1,2^, Sue Walters, BA^1^, Paola Chivers, PhD^6,7^, Samantha J. Winter, PhD^1,6^, Frank L. Mastaglia, MBBS, MD, FRACP^1,2^ and Ryan S. Anderton, PhD^1,2,6*^

^1^Perron Institute for Neurological and Translational Science, Nedlands

^2^ Centre for Neuromuscular and Neurological Disorders, University of Western Australia, Nedlands, Western Australia, Australia.
^3^ School of Biological Sciences, University of Western Australia, Crawley

^4^ Florey Institute for Neuroscience and Mental Health, University of Melbourne, Parkville Victoria, Australia.

^5^Centre for Clinical Neurosciences and Neurological Research, St Vincent’s Hospital Melbourne, Fitzroy, Victoria, Australia.
^6^ Institute for Health Research and School of Health Sciences, University of Notre Dame Australia, Fremantle, Western Australia, Australia.

^7^ Exercise Medicine Research Institute & School of Medical and Health Sciences, Edith Cowan University, Joondalup, Western Australia, Australia.

***Author correspondence:** Dr Ryan Anderton, School of Health Sciences, University of Notre Dame Australia, 19 Mouat Street, Fremantle, WA 6959, Australia
Tel: *+61 8 9433 0670*; Email: ryan.anderton@nd.edu.au

**Supplementary data**

**Supplementary Table 1.** Individual generalized linear models assessing cross-sectional associations between participant cognitive scores and clinical characteristics.

| **Outcome** | **Model** | **Naïve** | | | |
| --- | --- | --- | --- | --- | --- |
|  |  | **Intercept** | **β-Coefficient** | **SE** | ***p^b^* value** |
| ACE-R total score | Age at assessment | 109.239 | -0.314 | 0.058 | **<.001** |
| ACE-R total score | Age at onset | 96.770 | -0.138 | 0.054 | **.010** |
| ACE-R total score | Disease duration | 91.979 | -0.389 | 0.096 | **<.001** |
| ACE-R total score | LEDD | 90.517 | -0.002 | 0.001 | **.049** |
| ACE-R total score | No DBS* | 85.846 | 3.388 | 1.818 | .062 |
| Attention & orientation | Age at assessment | 19.146 | -0.028 | 0.009 | **.002** |
| Attention & orientation | Age at onset | 17.518 | -0.003 | 0.008 | .739 |
| Attention & orientation | Disease duration | 17.878 | -0.065 | 0.014 | **<.001** |
| Attention & orientation | LEDD | 17.610 | -3.1E-4 | 1.5E-4 | **.041** |
| Attention & orientation | No DBS* | 16.795 | 0.638 | 0.266 | **.016** |
| Memory score | Age at assessment | 29.960 | -0.119 | 0.020 | **<.001** |
| Memory score | Age at onset | 26.049 | -0.067 | 0.019 | **<.001** |
| Memory score | Disease duration | 23.061 | -0.101 | 0.034 | **.003** |
| Memory score | LEDD | 22.530 | -3.9E-4 | 3.4E-4 | .256 |
| Memory score | No DBS* | 20.923 | 1.483 | 0.631 | **.019** |
| Fluency score | Age at assessment | 13.367 | -0.052 | 0.018 | **.004** |
| Fluency score | Age at onset | 10.682 | -0.012 | 0.017 | .467 |
| Fluency score | Disease duration | 10.746 | -0.098 | 0.029 | **.001** |
| Fluency score | LEDD | 10.142 | -2.3E-4 | 3.1E-4 | .455 |
| Fluency score | No DBS* | 8.744 | 1.375 | 0.558 | **.014** |
| Language score | Age at assessment | 27.576 | -0.043 | 0.011 | **<.001** |
| Language score | Age at onset | 26.084 | -0.022 | 0.009 | **.022** |
| Language score | Disease duration | 25.144 | -0.041 | 0.018 | **.019** |
| Language score | LEDD | 25.206 | 4.3E-4 | 1.7E-4 | **.011** |
| Language score | No DBS* | 24.897 | -0.092 | 0.328 | .780 |
| Visuospatial-perceptual score | Age at assessment | 17.642 | -0.046 | 0.014 | **.001** |
| Visuospatial-perceptual score | Age at onset | 15.291 | -0.011 | 0.013 | .374 |
| Visuospatial-perceptual score | Disease duration | 15.344 | -0.087 | 0.022 | **<.001** |
| Visuospatial-perceptual score | LEDD | 15.161 | -0.001 | 2.2E-4 | **.013** |
| Visuospatial-perceptual score | No DBS* | 14.179 | 0.534 | 0.419 | .203 |

^b^*p* value taken from GLM without correction for covariates.

*Comparison category set to zero.

β, beta; SE, standard error; LEDD, Levodopa Equivalent Daily Dose; DBS, Deep Brain Stimulation; GLM, generalized linear models.

**Supplementary Table 2.** Akaike’s information criterion (AIC) Goodness of Fit

| Cognitive domain | AKAKIE naive - model summary | AKAKIE corrected - model summary |
| --- | --- | --- |
| **Total ACE-R** | 2936.277 | 2563.292 |
| **Attention & orientation** | 1446.754 | 1319.355 |
| **Memory** | 2113.445 | 2067.591 |
| **Fluency** | 2015.150 | 1993.720 |
| **Language** | 1602.806 | 1411.929 |
| **Visuospatial** | 1796.783 | 1607.229 |

ACE-R, Addenbrooke's Cognitive Examination – Revised

**Supplementary Table 3.** Individual generalized linear mixed models assessing longitudinal association between participant cognitive scores and clinical characteristics.

| **Outcome** | **Model** | **Naïve** | | | | |
| --- | --- | --- | --- | --- | --- | --- |
|  |  | **Intercept** | **β-Coefficient** | **SE** | **t value** | ***p^b^* value** |
| ACE-R total score | Years between follow-up | 89.808 | -0.534 | 0.321 | -1.661 | .098 |
| ACE-R total score | Age at assessment | 114.599 | -0.396 | 0.057 | -6.882 | **<.001** |
| ACE-R total score | Age at onset | 104.507 | -0.270 | 0.053 | -5.099 | **<.001** |
| ACE-R total score | Disease duration | 90.607 | -0.176 | 0.106 | -1.655 | .099 |
| ACE-R total score | LEDD | 89.151 | 2.4E-4 | 0.001 | 0.155 | .877 |
| ACE-R total score | No DBS* | 88.639 | 0.669 | 2.011 | 0.333 | .740 |
| Attention & orientation | Years between follow-up | 17.371 | -0.059 | 0.048 | -1.245 | .214 |
| Attention & orientation | Age at assessment | 19.707 | -0.038 | 0.009 | -3.941 | **<.001** |
| Attention & orientation | Age at onset | 18.884 | -0.028 | 0.009 | -3.277 | **.001** |
| Attention & orientation | Disease duration | 17.349 | -0.006 | 0.017 | -0.386 | .700 |
| Attention & orientation | LEDD | 17.130 | 2.1E-4 | 1.5E-4 | 1.400 | .163 |
| Attention & orientation | No DBS* | 17.361 | -0.069 | 0.310 | -0.223 | .824 |
| Memory score | Years between follow-up | 21.918 | -0.216 | 0.132 | -1.635 | .103 |
| Memory score | Age at assessment | 32.359 | -0.167 | 0.025 | -6.642 | **<.001** |
| Memory score | Age at onset | 27.971 | -0.112 | 0.023 | -4.810 | **<.001** |
| Memory score | Disease duration | 22.272 | -0.079 | 0.046 | -1.725 | .086 |
| Memory score | LEDD | 21.623 | 3.8E-5 | 4.2E-4 | 0.090 | .928 |
| Memory score | No DBS* | 20.322 | 1.479 | 0.859 | 1.722 | .086 |
| Fluency score | Years between follow-up | 10.486 | 0.105 | 0.090 | 1.160 | .247 |
| Fluency score | Age at assessment | 15.998 | -0.082 | 0.019 | -4.257 | **<.001** |
| Fluency score | Age at onset | 13.544 | -0.051 | 0.018 | -2.907 | **.004** |
| Fluency score | Disease duration | 11.047 | -0.047 | 0.033 | -1.405 | .161 |
| Fluency score | LEDD | 10.504 | 2.0E-4 | 3.0E-4 | 0.645 | .520 |
| Fluency score | No DBS* | 10.502 | 0.175 | 0.616 | 0.285 | .776 |
| Language score | Years between follow-up | 25.073 | -0.183 | 0.076 | -2.416 | **.016** |
| Language score | Age at assessment | 27.485 | -0.039 | 0.011 | -3.700 | **<.001** |
| Language score | Age at onset | 26.634 | -0.029 | 0.009 | -3.035 | **.003** |
| Language score | Disease duration | 25.062 | -0.011 | 0.019 | -0.544 | .587 |
| Language score | LEDD | 25.144 | -2.0E-4 | 1.7E-4 | -1.167 | .244 |
| Language score | No DBS* | 25.173 | -0.204 | 0.368 | -0.554 | .580 |
| Visuospatial-perceptual score | Years between follow-up | 14.934 | -0.160 | 0.071 | -2.257 | **.025** |
| Visuospatial-perceptual score | Age at assessment | 19.605 | -0.076 | 0.014 | -5.508 | **<.001** |
| Visuospatial-perceptual score | Age at onset | 17.667 | -0.052 | 0.013 | -4.109 | **<.001** |
| Visuospatial-perceptual score | Disease duration | 14.998 | -0.033 | 0.025 | -1.322 | .187 |
| Visuospatial-perceptual score | LEDD | 14.592 | 1.9E-4 | 2.3E-4 | 0.854 | .394 |
| Visuospatial-perceptual score | No DBS* | 15.344 | -0.663 | 0.462 | -1.435 | .152 |

^b^*p* value taken from GLMM without correction for covariates.

*Comparison category set to zero.

β, beta; SE, standard error; LEDD, Levodopa Equivalent Daily Dose; DBS, Deep Brain Stimulation; GLMM, generalized linear mixed models.

**Supplementary Table 4.** Akaike’s information criterion (AIC) Goodness of Fit

| Cognitive domain | AKAKIE naïve - model summary | AKAKIE corrected - model summary |
| --- | --- | --- |
| **Total ACE-R** | 1868.144 | 1823.064 |
| **Attention & orientation** | 931.509 | 922.970 |
| **Memory** | 1443.177 | 1401.292 |
| **Fluency** | 1284.476 | 1268.829 |
| **Language** | 1063.966 | 1054.611 |
| **Visuospatial** | 1131.943 | 1106.828 |

ACE-R, Addenbrooke's Cognitive Examination – Revised.
